# Prompt recurrence of granuloma annulare upon Janus kinase inhibitor discontinuation

**DOI:** 10.1016/j.jdcr.2026.04.054

**Published:** 2026-05-05

**Authors:** Arman Haveric, Patricia Zhao, Christopher D. Markeson, Sylvia Hsu

**Affiliations:** Department of Dermatology, The Lewis Katz School of Medicine at Temple University, Philadelphia, Pennsylvania

**Keywords:** granuloma annulare, JAK inhibitor, upadacitinib

## Introduction

Granuloma annulare (GA) is a benign granulomatous skin disorder of unknown etiology. The incidence of GA is 0.04% in the United States.^1^ Clinical subtypes of GA include localized, generalized, and subcutaneous variants. The pathogenesis of GA is not well-understood, and management is limited by a lack of evidence-based therapies.

There is currently no FDA-approved treatment for GA. Topical or intralesional corticosteroids have historically been employed as first-line treatment for localized disease while dapsone, phototherapy, hydroxychloroquine, and tumor necrosis factor inhibitors have been used to treat widespread disease.^1^ Recent reports have identified Janus kinase (JAK) inhibitors as promising off-label therapy for refractory GA, which is supported by emerging literature implicating the JAK-STAT pathway in the pathogenesis of GA.^2^^,^^3^ However, data on recurrence rates following treatment cessation are extremely limited. Here, we present a case of generalized GA, which fully cleared with the oral JAK inhibitor upadacitinib but which subsequently recurred upon medication cessation.

## Case presentation

A 46-year-old woman with a history of gastroesophageal reflux disease presented for evaluation of an eruption that had been present for over 1 year. She had previously tried clobetasol and tacrolimus ointments, prescribed by an outside dermatologist, with no improvement.

**Fig 1:**
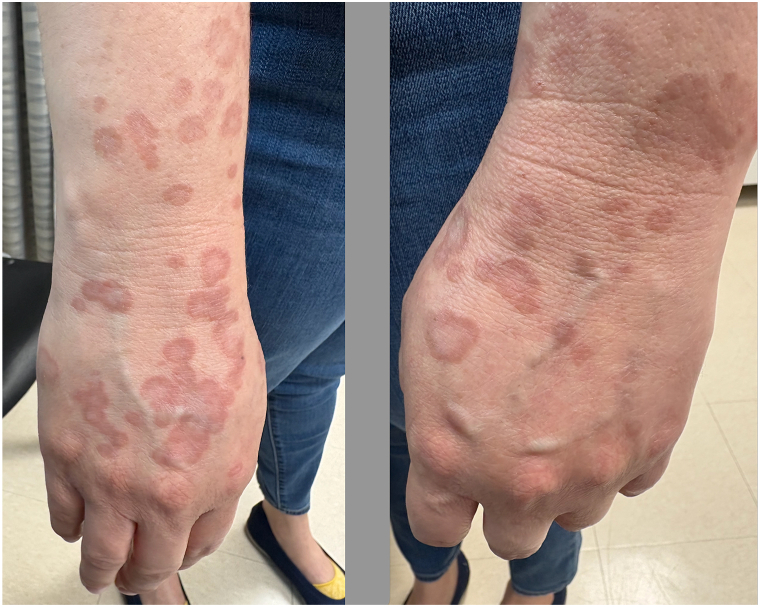
Annular, nonscaly, *dull red* plaques of granuloma annulare on dorsal hands.

Examination revealed annular, dull-red plaques on the face, chest, and bilateral upper extremities. The eruption was particularly striking on the dorsal hands (Fig 1). Based on the physical examination, a clinical diagnosis of GA was made. Given that the patient had already failed topical corticosteroid and calcineurin inhibitor therapy, JAK inhibitor therapy was pursued.

**Fig 2:**
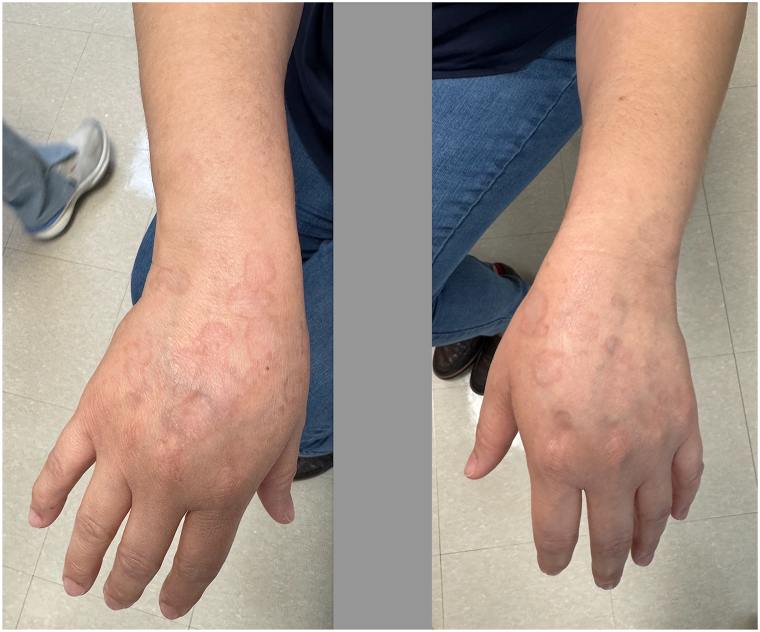
Granuloma annulare after 3 mo of upadacitinib 15 mg daily.

**Fig 3:**
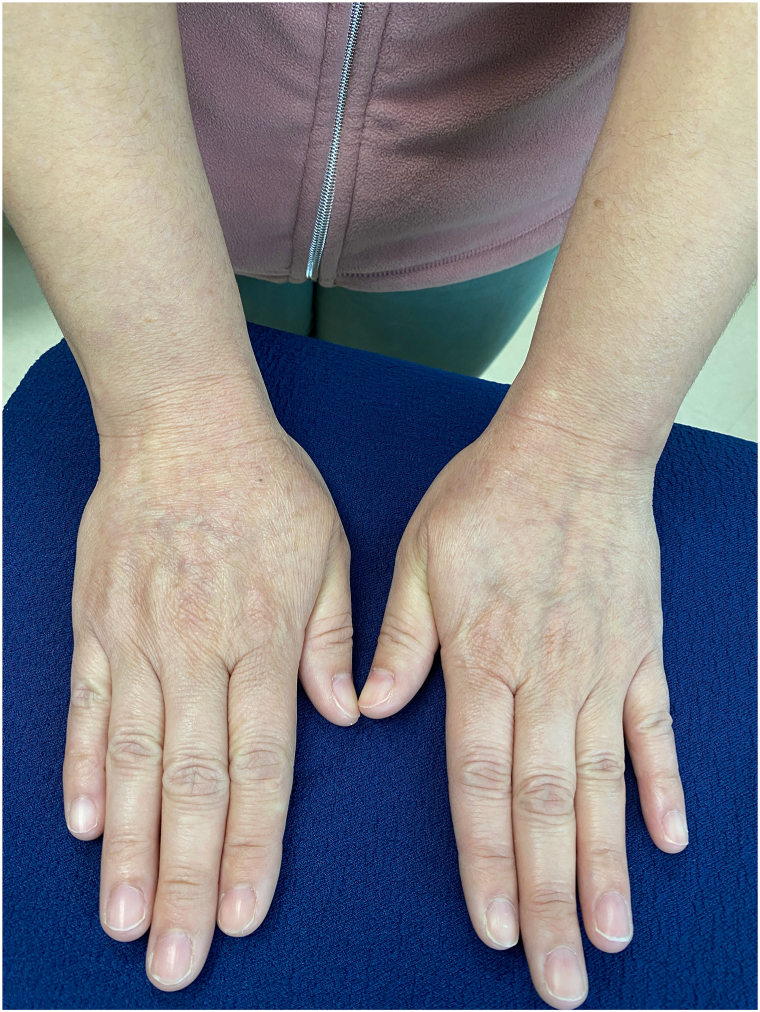
Clearance of granuloma annulare after 3 mo of upadacitinib 30 mg daily.

**Fig 4:**
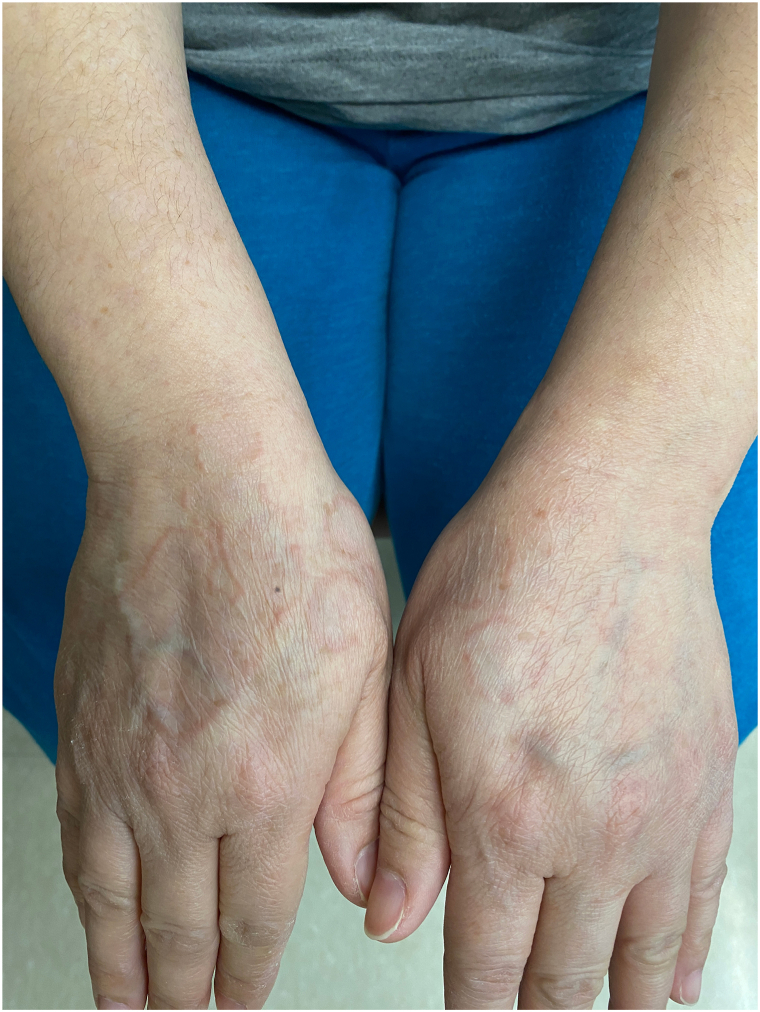
Recurrence of granuloma annulare 1 mo after discontinuing upadacitinib.

**Fig 5:**
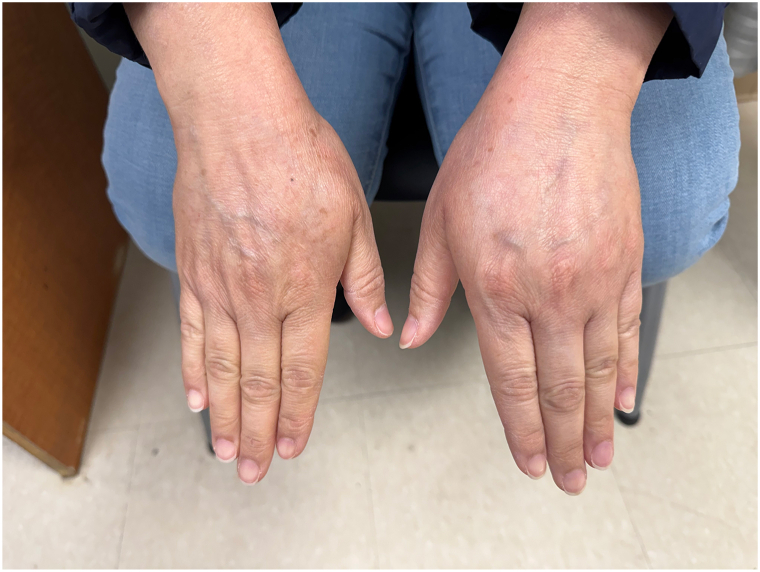
Clearance of granuloma annulare 2 mo after restarting upadacitinib 30 mg daily.

The patient was started on upadacitinib 15 mg daily, with significant improvement within 1 month. However, after 3 months, her dose was increased to 30 mg daily because she was not completely clear and had experienced eruption of new lesions (Fig 2). The patient returned for follow-up after 3 months at the higher dosage with complete resolution of her GA (Fig 3). At this point, upadacitinib was held to evaluate for sustained remission. Unfortunately, the eruption recurred within 2 weeks off JAK inhibitor therapy (Fig 4). As such, she was restarted on upadacitinib 30 mg daily. She redemonstrated fading of her GA lesions after 1 month, and full resolution after 2 months (Fig 5).

## Discussion

JAK inhibitors—including abrocitinib, upadacitinib, tofacitinib, baricitinib, and ruxolitinib—have been used off-label for GA with well-documented therapeutic success across numerous case series and review articles.^4^, ^5^, ^6^ Existing literature, however, has focused predominantly on outcomes during active treatment. Data on disease recurrence following JAK inhibitor discontinuation remain scarce.

The only comprehensive existing study documenting recurrence of GA lesions following JAK inhibitor cessation is a 2025 narrative review by Heidari et al,^5^ which identified 22 research studies (primarily case reports) covering 49 patients treated with JAK inhibitors for GA. Most patients had recalcitrant generalized GA, as in this case. The largest subset (29 patients) in the review was treated with tofacitinib, followed by upadacitinib (10), baricitinib (7), abrocitinib (2), and ruxolitinib (1). Complete or near-complete lesion resolution was reported in the majority (20) of studies. Follow-up data after JAK inhibitor cessation were reported in 31 patients (63%). Among patients with follow-up data, disease recurrence following medication cessation was reported in only 4 patients across 3 studies: 2 patients taking baricitinib^7^^,^^8^ and 2 taking tofacitinib.^9^ (Table I) No studies evaluated upadacitinib specifically. Time to recurrence was variable, ranging from 3 days to 3 months. Age, sex, and prior medical history were reported for only 2 of 4 patients. Generalized GA was the reported subtype in all cases.

**Table I:** Case reports documenting recurrence of granuloma annulare lesions following cessation of Janus kinase inhibitor therapy

Reference	Medication	Past medical history	Age	Sex	GA subtype	Outcome following cessation	Maintenance therapy following recurrence	Follow-up window on maintenance therapy	Clinical outcome on maintenance therapy
Jadoul et al^7^	Baricitinib 4 mg once daily	Obesity, HTN, multinodular goiter, hemochromatosis	66	F	Generalized	Severe flare after cessation	2 mg once daily	8 mo	Clinical improvement
Kim et al^8^	Baricitinib 4 mg once daily	T2DM, HTN	67	F	Generalized	Lesion recurrence 3 d after cessation	2-4 mg once daily	NS	NS
Dev et al^9^	Tofacitinib 5 mg twice daily	NS	NS	NS	Generalized	Lesion recurrence 1 mo after cessation	5 mg twice daily	1 mo	Resolution
Dev et al^9^	Tofacitinib 5 mg twice daily	NS	NS	NS	Generalized	Lesion recurrence 3 mo after cessation	5 mg twice daily	1 mo	Resolution

*GA*, Granuloma annulare; *HTN*, hypertension; *T2DM*, type 2 diabetes mellitus.

The paucity of post-treatment follow-up data for patients on JAK inhibitor therapy has not been systematically reported in the literature, likely reflecting the recency of JAK inhibitor use for GA. Our case highlights the need for future research on maintenance dosing and tapering protocols, as literature to date has been focused on clinical efficacy during active treatment but not on outcomes following cessation.^4^ Female sex was the only commonality among reported cases^7^^,^^8^ and our case. Medical history, age, and time to recurrence were otherwise variable.

Future research could better characterize clinical and demographic factors associated with lesion recurrence, during active treatment or after treatment cessation. Additionally, the superiority of different oral JAK inhibitors in the treatment of GA has not yet been demonstrated due to lack of comparative clinical trial data.^4^^,^^5^ Research is needed to clarify the differences in efficacy and safety profiles of different oral JAK inhibitors for refractory GA, and to evaluate for long-term disease control following medication taper or cessation.

## Key takeaways from this case and the existing literature

• Gap in postcessation data: Recurrence and follow-up data are missing for a substantial proportion of patients in published JAK inhibitor studies for GA, limiting the ability to counsel patients on long-term prognosis.
• Rapid recurrence: Recurrence within 2 weeks of discontinuation—consistent with the baricitinib case reported by Kim et al^8^ (3 days)—suggests that JAK inhibitor therapy may be suppressive rather than disease-modifying for some patients.
• Need for future research: Studies are needed to define optimal maintenance dosing and tapering protocols.

## Conflicts of interest

None disclosed.
